# Supplementary material for: Active site specificity profiling datasets of matrix metalloproteinases (MMPs) 1, 2, 3, 7, 8, 9, 12, 13 and 14
Source: Data Brief. 2016 Feb 22;7:299–310. doi: 10.1016/j.dib.2016.02.036 (PMC4777984; doi:10.1016/j.dib.2016.02.036)
Supplement: Supplementary file 10 — Supplementary material [file mmc10.zip › WebPICS_hMMP9_G_1%/P1prime.html]

 

PICS results


|  |  |
| --- | --- |
| **P1prime\_C**  11 in 146 sites   7.5 %    effects > 10 perc. pnts.  (vice-versa in brackets)  P1\_D: 17.7 (13.9)   P1\_G: 22.0 (11.5)   P2prime\_A: 18.4 (15.6) |  |
| **P1prime\_F**  8 in 146 sites   5.5 %    effects > 10 perc. pnts.  (vice-versa in brackets)  P1\_G: 35.6 (13.5)   P1\_N: 16.8 (11.2)   P2prime\_L: 25.2 (11.2) |  |
| **P1prime\_I**  22 in 146 sites   15.1 %    effects > 10 perc. pnts.  (vice-versa in brackets)  P3\_A: 12.2 (12.2)   P2\_A: 14.3 (16.5)   P1\_K: 10.7 (21.3) |  |
| **P1prime\_L**  44 in 146 sites   30.1 %    effects > 10 perc. pnts.  (vice-versa in brackets)  P3prime\_S: 10.6 (15.1) |  |
| **P1prime\_Q**  12 in 146 sites   8.2 %    effects > 10 perc. pnts.  (vice-versa in brackets)  P3\_V: 16.1 (14.9)   P2\_Q: 12.6 (25.1)   P1\_A: 41.9 (21.0)   P2prime\_K: 29.4 (19.6)   P2prime\_Q: 20.9 (41.8) |  |
| **P1prime\_V**  12 in 146 sites   8.2 %    effects > 10 perc. pnts.  (vice-versa in brackets)  P2\_R: 19.6 (11.8)   P2prime\_I: 24.4 (22.6)   P3prime\_N: 12.6 (25.1) |  |
